# Effects of growth hormone in the central nervous system

**DOI:** 10.20945/2359-3997000000184

**Published:** 2019-11-01

**Authors:** Frederick Wasinski, Renata Frazão, Jose Donato

**Affiliations:** 1 Departamento de Fisiologia e Biofísica Instituto de Ciências Biomédicas Universidade de São Paulo São Paulo SP Brasil Departamento de Fisiologia e Biofísica, Instituto de Ciências Biomédicas, Universidade de São Paulo (USP), São Paulo, SP, Brasil; 2 Departamento de Anatomia Instituto de Ciências Biomédicas Universidade de São Paulo São Paulo SP Brasil Departamento de Anatomia, Instituto de Ciências Biomédicas, Universidade de São Paulo (USP), São Paulo, SP, Brasil

**Keywords:** GH, cytokine, brain, STAT5, metabolism

## Abstract

Growth hormone (GH) is best known for its effect stimulating tissue and somatic growth through the regulation of cell division, regeneration and proliferation. However, GH-responsive neurons are spread over the entire central nervous system, suggesting that they have important roles in the brain. The objective of the present review is to summarize and discuss the potential physiological importance of GH action in the central nervous system. We provide evidence that GH signaling in the brain regulates the physiology of numerous functions such as cognition, behavior, neuroendocrine changes and metabolism. Data obtained from experimental animal models have shown that disruptions in GH signaling in specific neuronal populations can affect the reproductive axis and impair food intake during glucoprivic conditions, neuroendocrine adaptions during food restriction, and counter-regulatory responses to hypoglycemia, and they can modify gestational metabolic adaptions. Therefore, the brain is an important target tissue of GH, and changes in GH action in the central nervous system can explain some dysfunctions presented by individuals with excessive or deficient GH secretion. Furthermore, GH acts in specific neuronal populations during situations of metabolic stress to promote appropriate physiological adjustments that restore homeostasis. Arch Endocrinol Metab. 2019;63(6):549-56

## INTRODUCTION

Growth hormone (GH) is the most abundant factor secreted by the anterior pituitary gland. GH secretion is controlled by hypothalamic neurons that secrete stimulatory or inhibitory neuropeptides into the hypophyseal portal system to regulate the synthesis and release of GH by somatotropic cells. In this regard, GH-releasing hormone (GHRH)-expressing neurons induce GH release, whereas somatostatin (SST)-expressing neurons have an inhibitory effect on pituitary GH secretion (1,2). However, other neuropeptides and hormonal factors also regulate GH secretion. For example, the hormone ghrelin (*Growth Hormone-RELeasINg peptide*) is mainly produced in the stomach, and systemic ghrelin administration induces robust GH secretion (2). In addition, ghrelin secretion is required for increasing GH levels during prolonged food restriction (3). However, during short-term fasting, ghrelin does not regulate GH secretion (4). Although ghrelin can cause a tonic increase in baseline GH levels during prolonged starvation (3), GH pulsatility is suppressed in fasting mice (5). During fasting, neuropeptide Y (NPY)-expressing neurons become active (6), and NPY secretion is responsible for decreasing GH pulsatility via the activation of the Y1 receptor (5). In addition to fasting, other conditions that induce increased GH secretion include hypoglycemia (7), physical exercise (8) and pregnancy (9).

The most well-known function of GH is related to tissue and somatic growth, which is realized through the regulation of cell division, regeneration and proliferation in various tissues (10). GH also possesses noteworthy metabolic effects. In this sense, GH secretion increases lipolysis and circulating free fatty acids. Accordingly, GH-deficient or GH receptor (GHR)-knockout animals frequently exhibit increased body adiposity, particularly in the subcutaneous adipose tissue (11). GH also has hyperglycemic effects, which are manifested either by stimulated hepatic gluconeogenesis (10,11) or increased insulin resistance in skeletal muscle (12). Therefore, high GH levels can cause insulin resistance and diabetes mellitus (13). Importantly, several effects of GH are mediated by another hormone, insulin-like growth factor-1 (IGF-1) (10). The activation of GHR induces IGF-1 expression in numerous tissues. IGF-1 can have a local autocrine or paracrine action, or IGF-1 can be secreted from the liver into the blood. Therefore, GH action in the liver regulates IGF-1 circulating levels (10). As a consequence, liver-specific GHR-knockout mice exhibit very low levels of circulating IGF-1 and reduced body weight and length (14). Of note, high GH levels are observed in liver-specific GHR-knockout mice (14), indicating that IGF-1 also regulates GH secretion via negative feedback loops, possibly involving the pituitary gland and hypothalamus (15). Therefore, the somatotropic axis can be summarized by the hypothalamic control of pituitary GH secretion, the activation of GHR in several tissues, and the hepatic secretion of IGF-1 that, together with GH, regulates several metabolic and growth functions. In the present review, our aim is to summarize and discuss the potential physiological roles of GH action in the central nervous system (CNS).

## THE BRAIN IS A GH-TARGET TISSUE

The most well-known biological effects of GH are mediated by the liver, white adipose tissue, skeletal muscle and bone (10). However, the brain also expresses GHRs (1). Brain GHR expression is important for enabling the neuroendocrine neurons to sense GH levels and regulate pituitary GH secretion via negative feedback (15). Accordingly, GHR is highly expressed in the arcuate nucleus of the hypothalamus (ARH), the site that contains the most GHRH neurons (1,16). In addition, approximately 70% of the SST neurons in the paraventricular and periventricular nuclei coexpress GHR mRNA (1). Central administration of antisense GHR mRNA decreases hypothalamic SST expression, which increases GH pulsatility, demonstrating the key role SST neurons play in mediating GH negative feedback (17). However, GH-responsive cells are not restricted to the hypothalamus, and several brain areas, including the septum, bed nucleus of the stria terminalis, thalamus, amygdala, hippocampus and brainstem, also express GHRs (1,16,18).

**Figure 1:**
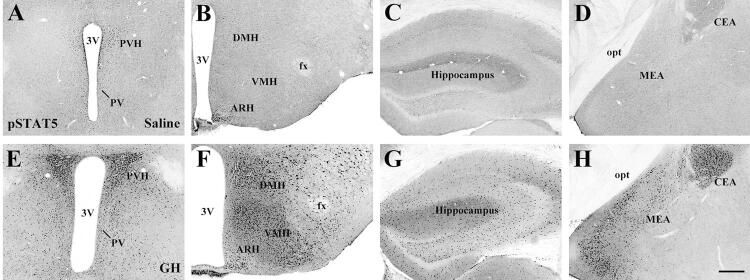
Distribution of GH responsive cells in different brain areas of mice via the detection of STAT5 phosphorylation (pSTAT5). A-H. Brightfield photomicrographs of brain sections showing the distribution of pSTAT5 immunoreactive cells in saline-injected mice (A-D) or in GH-injected mice (E-H). Mice received an intraperitoneal injection of 20 µg of porcine GH per gram of body weight, followed by perfusion 90 minutes later. Abbreviations: 3V, third ventricle; ARH, arcuate nucleus; CEA, central nucleus of the amygdala; DMH, dorsomedial nucleus; fx, fornix; MEA, medial nucleus of the amygdala; opt, optic tract; PVH, paraventricular nucleus; VMH, ventromedial nucleus. Scale Bar = 200 µm. Figure adapted from the study by Furigo and cols. (47).

GHR is a member of the type I cytokine receptor family and relies mostly on the janus kinase 2 (JAK^2^)/signal transducer and activator of transcription (STAT) pathway to induce its intracellular effects (10). Although the activation of GHR recruits several intracellular signaling molecules, including STAT1, STAT3 and Src kinases (10), STAT5 is considered the most relevant intracellular pathway induced by GHR activation. Accordingly, STAT5-knockout mice exhibit reduced growth similar to that caused by GHR deficiency (19). Since classical methods to detect GHR do not enable good resolution at the cellular level, our group employed an alternative strategy to identify GH-responsive cells in the mouse brain (18). Using this technique, mice receive acute systemic injection of GH, and after a sufficient time for GH to act throughout the body, the brain is histologically processed to detect STAT5 phosphorylation (pSTAT^5^). Thus, pSTAT5 can be used as a marker of GH-responsive cells in the mouse brain (Figure 1). Using this approach, we confirmed the distribution of GH-responsive cells in brain areas previously shown to express GHR and further demonstrated that GH-responsive cells are widely distributed in numerous structures of the CNS (Figure 1). Therefore, not only the brain is a GH-target tissue, but the large number of GH-responsive cells in brain structures involved with different functions suggest that central GH action is likely more relevant than previously thought.

## COGNITIVE EFFECTS OF GH

**Figure 2:**
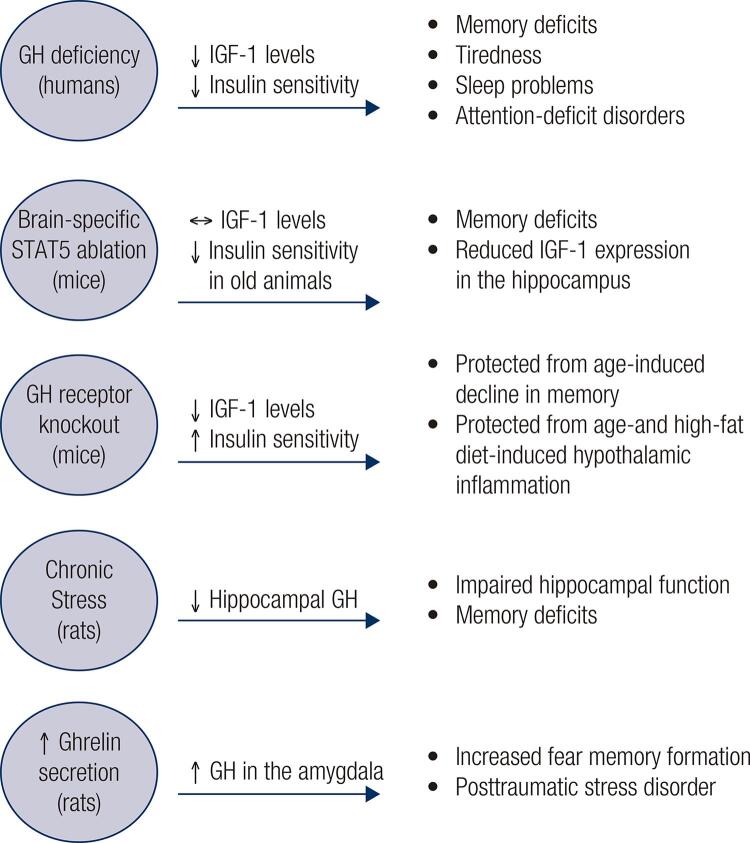
Scheme that summarizes the cognitive effects induced by GH signaling in the brain.

Previous reports indicate that GH-deficient individuals may exhibit several cognitive and neurological abnormalities, including poor memory, tiredness, sleep problems, decreased well-being and mood and attention-deficit disorders (Figure 2). Additionally, GH has neuroprotective effects, and some cognitive consequences of aging are possibly associated with the decrease in GH secretion typical of elderly people (20). However, although GH deficiency is an interesting model to use for determining the role of GH on cognitive functions, GH-deficient individuals present multiple endocrine and metabolic abnormalities, making it difficult to determine whether the effects are caused by the lack of GHR signaling *per se* or by a secondary, indirect factor. The major confounding factor associated with GH deficiency is decreased circulating IGF-1 levels (Figure 2) since IGF-1 also has neuroprotective effects (21) and regulates the expression of molecular factors involved in cognitive functions (22). Moreover, brain insulin resistance is associated with IGF-1 resistance, and these defects contribute to the cognitive decline in patients with Alzheimer’s disease (23,24).

Since the activation of GHR, but not of IGF-1 receptor, recruits the STAT5 signaling pathway, manipulating this transcription factor may be an alternative way to study the effects of GH on the brain, regardless of circulating IGF-1 (25). Thus, our group generated a brain-specific STAT5-knockout mouse (25). A previous study had shown that these mice have normal body growth and serum IGF-1 levels, although brain-specific STAT5-knockout mice exhibit late onset obesity and insulin resistance (26). Importantly, GH action in the brain is impaired in these mice since the major intracellular pathway recruited by GHR is nonfunctional. We observed that brain-specific STAT5 ablation leads to impaired learning and memory formation, as determined by results from the novel object recognition test, Barnes maze and fear conditioning test (25). Although hippocampal neurogenesis is normal (25), these mutant mice exhibit decreased IGF-1 expression in the hippocampus, suggesting that GH-mediated hippocampal IGF-1 production is important for maintaining memory (Figure 2). Corroborating the idea that GH directly regulates neuronal aspects in the brain, GHR-knockout mice have suppressed development of the projections that extend from ARH neurons to target areas (27). Importantly, these projections are normal in liver-specific GHR-knockout mice despite their reduced circulating IGF-1 levels (27). Thus, the development of ARH neuronal projections seems to be regulated by GH signaling, regardless of circulating IGF-1 levels.

Although GH-deficient individuals frequently present impaired memory and other cognitive abnormalities (20), GHR-knockout mice are protected from age-induced decline in memory retention, possibly because of changes in glutamatergic neurotransmission in the hippocampus (28,29). GHR-knockout mice are also protected from age- and high-fat-induced hypothalamic inflammation (27,30). The “paradoxical” cognitive improvement and protection against hypothalamic inflammation in aged GHR-knockout mice may be related to the higher insulin sensitivity observed in these animals (11) as insulin resistance has been linked with impaired cognition and is a risk factor for Alzheimer’s disease (23,24). Accordingly, 12-month-old transgenic mice that overexpress a GHR antagonist exhibited improved insulin sensitivity and learning, whereas overexpressed GH caused insulin resistance and impaired memory retention (31). Thus, these studies indicate that excess GH has a negative impact on cognition, while inhibition of GH action can improve spatial learning and memory during aging, even though these effects may be secondary to changes in insulin sensitivity (Figure 2).

GH can also be synthesized by brain cells, including hippocampal neurons. Chronic stress causes a decrease in hippocampal GH levels and impairs hippocampal function, including memory and learning (32). Restoration of hippocampal GH reverses stress-related impairments promoting stress resilience (32). In contrast, upregulation of GH in the amygdala increases the number of cells activated by fear memory formation (33). Virus-mediated overexpression of GH in the amygdala increases fear, an effect also observed in ghrelin-treated animals (34). Thus, ghrelin-induced GH expression in the amygdala may be involved in maladaptive changes following prolonged stress, such as posttraumatic stress disorder (33,34). Notably, our group showed that both the hippocampus and several areas of the amygdala, including the medial and central nuclei, contain a large number of GH responsive cells and therefore functional GHR expression (18) (Figure 1).

## GH MODULATES THE HYPOTHALAMIC-PITUITARY-GONADAL (HPG) AXIS

Among the multiple factors that influence reproduction, GH is a hormonal component required for sexual maturation and sex steroid mediation of the ovulatory cycle (15). GH secretion affects estradiol synthesis, whereas puberty also modifies GH release (35). GH therapy can accelerate puberty onset in healthy individuals and restore the time of puberty and fertility in GH-deficient women or GH-deficient dwarf mice. Furthermore, GH therapy can improve the sensitivity of the ovaries to gonadotropin stimulation in women being treated for fertility-related problems (35-38). Although GH acts directly in the gonads, this effect is not sufficient to explain the late onset puberty, the lack of sexual maturation and the infertility found in individuals with GH deficiency or resistance (37,39). Accordingly, we recently described that critical neuronal populations that regulate the HPG axis are responsive to GH, including neurons of the anteroventral periventricular and rostral periventricular nuclei (AVPV/PeN), ventral premammillary nucleus (PMv) and ARH (18,40). These areas contain either kisspeptin or leptin receptor (LepR)-expressing neurons and are required for sexual maturation and the maintenance of fertility (41,42).

The effects of GH on the central components of the HPG axis are likely mediated by the JAK2/STAT5 signaling pathway. In this sense, while a systemic GH injection induces STAT5 phosphorylation in AVPV/PeN kisspeptin neurons or PMv cells, a GHR agonist does not acutely change the resting membrane potential of the hypothalamic kisspeptin, PMv or GnRH neurons, an effect that would require the modulation of membrane ion channels by fast-acting signaling pathways (40,43). Interestingly, ARH kisspeptin neurons do not express GH-induced pSTAT5, indicating that only kisspeptin neurons of the AVPV/PeN are directly responsive to GH (40).

To further understand the physiological relevance of GH signaling in the central components of the HPG axis, we ablated GHR in kisspeptin cells, LepR-expressing cells and in the entire brain. GHR ablation in the kisspeptin cells caused a reduction in the hypothalamic expression of genes related to the reproductive axis in pubertal female mice, including the *Gnrh1*, *Kiss1*, *Nos1* and *Esr1* (44). Despite these changes, GH signaling in kisspeptin cells was not required for sexual maturation or the sex steroid mediation of the ovulatory cycle (44). In contrast, ablation of GHR in the LepR-expressing cells was associated with lower body weight and reduced serum leptin levels during development, which led to delayed sexual maturation. Moreover, some individuals presented disruption in the estrous cycle (44). Ablation of the GHR from the entire brain disrupted GH negative feedback in the hypothalamus, leading to increased GH secretion. Augmented GH secretion was accompanied by increased uterine mass and lower serum leptin levels (44). Despite these effects, the brain-specific GHR-knockout mice underwent puberty at the normal time (44). These findings suggest that GH can modulate brain components of the HPG axis, although central GH action is not required for the timing of puberty. On the other hand, the metabolic alterations induced by the disruption of GH signaling may indirectly affect sexual maturation and the estrous cycles in adult animals.

## GH ACTION IN THE CNS REGULATES METABOLISM

**Figure 3:**
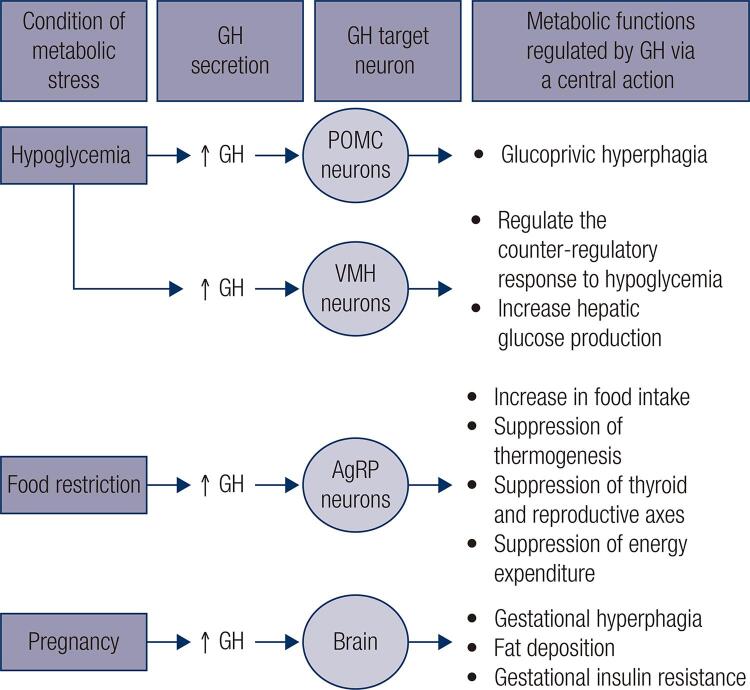
GH acts in specific neuronal populations during situations of metabolic stress to promote appropriate physiological adjustments in order to restore the homeostasis.

As mentioned earlier, GH has marked metabolic actions; in particular, GH has a lipolytic effect and induces insulin resistance (10,11,13). GH also causes metabolic changes in the organism through its action in the CNS (Figure 3). GH overexpression in the brain increases food intake in mice and carps, causing obesity (45,46). An intracerebroventricular administration of GH increases food intake 24 hours after the injection (47). Thus, GH produces an orexigenic response in the CNS. In an evolutionary perspective, it is advantageous to link the growth-promoting effects of GH with higher food intake to provide the nutrients needed for growth.

Recent studies have started to unravel the neural pathways recruited by GH to modulate metabolism (Figure 3). The orexigenic effect of GH is possibly induced by ARH neurons that coexpress NPY and the agouti-related peptide (AgRP). First, it is well known that ARH AgRP/NPY neurons are potent inducers of food intake (6). Second, 95% of the AgRP/NPY neurons in the ARH exhibit either GHR expression (48) or GH-induced pSTAT5 (47). Third, acute GH injection increases the hypothalamic expression of *Agrp* and *Npy* transcripts, and GH induces significant excitation in 25% of the ARH AgRP/NPY neurons, depolarizing their resting membrane potential and increasing the frequency of the action potential (47). Finally, ghrelin also stimulates food intake via ARH AgRP/NPY neurons (49). Of note, GHR-knockout mice are unresponsive to the orexigenic effect of ghrelin, suggesting that GH signaling is required for the effects of ghrelin on food intake (50). Therefore, these studies indicate that ARH AgRP/NPY neurons are important mediators of the orexigenic effects of GH.

To determine the importance of GH action on the ARH AgRP/NPY neurons for the regulation of energy homeostasis, our group produced mice carrying a specific ablation of GHR only in AgRP neurons (47). AgRP-specific GHR ablation caused no changes in body weight, body composition, food intake, energy expenditure or glucose homeostasis in male or female mice (47,51). However, when these mice were exposed to food restriction, the AgRP-specific GHR-knockout mice were unable to present the typical neuroendocrine adaptations to this condition. In this sense, while the control animals suppressed the thermogenic markers in the brown adipose tissue (BAT) and thyroid and reproductive axes during food restriction, the AgRP-specific GHR-knockout mice sustained high levels of circulating T4 and testosterone and had unchanged uncoupling protein 1 expression in their BAT compared to *ad libitum* fed animals (47). Additionally, the increase in *Agrp* and *Npy* expression in the hypothalamus during food restriction was prevented in the AgRP-specific GHR-knockout mice. Consequently, the control animals progressively decreased their energy expenditure during food restriction, whereas the AgRP-specific GHR-knockout mice maintained a higher energy expenditure, which led to increased weight loss during food restriction compared to the weight changes in the control mice (47). Thus, this study revealed a new biological function of GH: it alerts ARH AgRP/NPY neurons about food restriction. Therefore, these neurons can regulate energy expenditure accordingly (Figure 3). Although these adaptions had great evolutionary value, possibly increasing the chances of survival in times of famine, these energy-saving adaptions hinder the treatment of obesity by reducing the energy expenditure (52). Thus, the identification of the factors that cause these metabolic alterations during food restriction may potentially help in the development of more efficient therapeutic approaches for the treatment of obesity. In this regard, our group showed that administration of pegvisomant, a GHR antagonist, is able to increase the energy expenditure of food-deprived wild-type mice (47). A recent study also showed that plasma AgRP levels in humans, which can be used as a marker of hypothalamic AgRP expression, exhibit a positive correlation with circulating GH and IGF-1 levels (53). Individuals with acromegaly have high plasma AgRP levels and AgRP concentration decreases after surgical or pegvisomant treatment (53). These findings provide additional evidence in humans that AgRP neurons mediate the effects of GH on energy metabolism.

In the ARH, GH responsive cells are not restricted to AgRP/NPY neurons. In another study, we showed that approximately 60% of the neurons that express pro-opiomelanocortin (POMC) in the ARH express pSTAT5 after an injection of GH (54). GH action in POMC cells is not necessary for the regulation of energy or glucose homeostasis under basal conditions. However, the hyperphagia induced by a glucoprivic situation, caused by the administration of 2-deoxy-D-glucose (a drug that causes glucopenia and induces a robust counter-regulatory response), is significantly attenuated in mice lacking GHR in their POMC cells (Figure 3), indicating that GH action in POMC neurons regulates food intake in specific situations (54). GH is robustly secreted during hypoglycemia (7), and GH deficiency causes spontaneous hypoglycemia and impairs the counter-regulatory response to hypoglycemia (3,55). However, the mechanisms activated by GH that contribute to the recovery of hypoglycemia are unknown. In a recent study (56), we showed that a large number of GH-responsive neurons are found in the ventromedial nucleus of the hypothalamus (VMH), a key relay station that regulates the counter-regulatory response to hypoglycemia (57). Although ablation of GHR in VMH neurons did not affect glucose tolerance or insulin sensitivity, the absence of GH action in the VMH neurons impaired the capacity of the mice to recover from insulin-induced hypoglycemia and significantly decreased the counter-regulatory response induced by 2-deoxy-D-glucose injection (56). The effects of GH regulation on the counter-regulatory response are likely mediated by the parasympathetic nervous system. In this sense, infusion of 2-deoxy-D-glucose produced abnormal hyperactivity in the parasympathetic preganglionic neurons in mice with GHR-ablated VMH neurons. In addition, pharmacological blockers of the parasympathetic nervous system prevent the counter-regulatory defects caused by GHR ablation in the VMH (56). Unlike the effects induced by GHR depletion in the POMC neurons (54), GHR-ablated VMH neurons did not affect glucoprivically induced hyperphagia (56), demonstrating that GH action in each neuronal population induces very specific effects (Figure 3).

One aspect shared between AgRP/NPY, POMC and VMH neurons is the expression of LepR. In addition, the action of leptin in these neuronal populations is important for the regulation of energy and glucose homeostasis (6). However, several other neuronal populations and peripheral cells also express the LepR (6). To determine whether GH acts on leptin responsive cells, Cady and cols*.* (58) studied mice carrying ablation of GHR in all LepR-expressing cells. These authors found that the lack of GH signaling in the LepR-expressing cells impaired hepatic insulin sensitivity and peripheral lipid metabolism (58). Since this phenotype is not observed in the mice with ablated GHR in the AgRP/NPY, POMC and VMH neurons, it is likely that other LepR-expressing cells mediate the insulin resistance of the mice with diminished GHR signaling in the LepR cells.

Pregnancy is a condition characterized by marked metabolic adaptations that prepare the maternal organism for the energy demands of the offspring. In this regard, pregnant animals usually increase their food intake, accumulate body fat and develop transient insulin resistance (59,60). These adaptations are thought to be mediated by hormones secreted during pregnancy, principally prolactin and placental lactogens (59). High amounts of GH are also secreted during pregnancy in both humans and rodents (9). In a recent study, we showed that ablation of GHR in the entire brain or in LepR-expressing cells improved the systemic insulin sensitivity of pregnant mice (51). Furthermore, inactivation of GHR in the brain led to decreased food intake and body adiposity during pregnancy, whereas depletion of GHR in the LepR cells increased leptin sensitivity of VMH neurons (51). These findings indicate that central GH action regulates energy and glucose homeostasis during pregnancy. Specifically, GH action in the brain partially mediates the increases in food intake, body adiposity and insulin resistance observed in pregnant mice (Figure 3).

## CONCLUDING REMARKS

In this review, we summarized the known effects of GH on the CNS. We provided evidence that the brain should be considered an important target of GH to regulate key physiological aspects such as cognitive, behavioral, neuroendocrine and metabolic functions. GH-responsive neurons are spread over virtually the entire CNS, suggesting that the importance of GH signaling in the brain is likely greater than previously thought. The role played by each specific neuronal population mediating the effects of GH is beginning to be revealed (Figure 3). However, additional studies are still necessary to determine other effects that may be mediated by GH via the CNS. The knowledge of the importance of the CNS for the effects of GH contributes to the understanding of the dysfunctions caused by excessive or deficient GH secretion, either in pathological conditions or in physiological situations (e.g., aging, pregnancy or food deprivation).
